# Genomic surveillance of SARS-CoV-2 in the Andean Community (2020–2024): integrating regional sequencing efforts from Colombia, Ecuador, Peru, and Bolivia through “ORAS-CONHU” program

**DOI:** 10.3389/fpubh.2025.1623413

**Published:** 2025-09-11

**Authors:** Alfredo Bruno, Doménica de Mora, Paola Rojas-Estevez, Héctor Alejandro Ruiz-Moreno, Jessica Guzman-Otazo, Omar Cáceres, Victor Alberto Jiménez Vásquez, Jimmy Garcés, Maritza Olmedo, Carlos Franco-Muñoz, Veronica Medrano Romero, Evelin Esther Fortun Fernandez, Maria Renee Castro Cusicanqui, Miguel Angel Garcia-Bereguiain, “Grupo Andino de Vigilancia Genómica de SARS-CoV-2”, María del Carmen Calle Dávila

**Affiliations:** ^1^Instituto Nacional de Salud Pública e Investigación "Leopoldo Izquieta Pérez”, Guayaquil, Ecuador; ^2^Universidad Agraria de Ecuador, Guayaquil, Ecuador; ^3^Grupo de Genómica de Microorganismos Emergentes, Dirección de Investigación en Salud Pública, Instituto Nacional de Salud, Bogotá, Colombia; ^4^Instituto Nacional de Laboratorios de Salud “Dr. Nestor Morales Villazón”, La Paz, Bolivia; ^5^Equipo de Vigilancia Genómica de SARS-CoV-2, Instituto Nacional de Salud, Lima, Peru; ^6^Grupo de Parasitología, Dirección de Investigación en Salud Pública, Instituto Nacional de Salud, Bogotá, Colombia; ^7^Ministerio de Salud y Deportes, La Paz, Bolivia; ^8^One Health Research Group, Universidad de Las Americas, Quito, Ecuador

**Keywords:** SARS-CoV-2, South America, genomic surveillance, COVID-19, Andean Community

## Abstract

**Introduction:**

The COVID-19 pandemic has deeply affected Latin American countries, with countless COVID-19 cases and deaths. In countries like Peru, Colombia, Ecuador and Bolivia there was a collapse of the public health system, and the lack of testing capacity did not allow to control the spread of SARS-CoV-2 during the first year of the COVID-19 pandemic. After these dramatic beginnings, regional efforts focused on improving testing capacity and massive vaccinations campaigns, but also implementing a sustained SARS-CoV-2 genomic surveillance program to follow up the evolution of the virus.

**Methods:**

This study examines the regional efforts in the Andean Community in terms of SARS-CoV-2 genomic surveillance for Colombia, Ecuador, Peru, and Bolivia in coordination with “Organismo Andino de Salud-Convenio Hipólito Unanue” (ORAS-CONHU). Moreover, SARS-CoV-2 emerging lineages distribution, clade determination and phylogenetic analysis in those countries for the period 2020–2024 was done by retrieving 16,867 sequences from the GISAID database.

**Results:**

From the initial lineages 19A and 19B, lineages 20A, 20B, and 20C emerged in 2020, followed by several variants such as 20 J (Gamma), 21A (Delta), 21G (Lambda), and 21H (Mu) emergence along 2021; by the end of 2022, the highly transmissible Omicron variants (21 K, 21 L) emerged and have been evolving into multiple sub variants lineages like 22F, 23A, and 23I (JN.1), the latest dominant along 2024.

**Discussion:**

While each country exhibits some specific characteristics, the phylogenetic analysis underscored a common pattern in the lineage evolution of SARS-CoV-2 in the Andean Community supporting a rapid transnational transmission of the virus. This study was part of a regional effort to develop an integrative transnational genomic surveillance network for SARS-CoV-2 as a proxy for a more ambitious regional infectious diseases genomic surveillance program.

## Introduction

The emergence of Coronavirus Disease 2019 (COVID-19), caused by Severe Acute Respiratory Syndrome Coronavirus 2 (SARS-CoV-2) has caused an unprecedented worldwide public health crisis leading to high fatality rates and healthcare systems collapse. After the first reports of a severe respiratory disease caused by a new virus in Wuhan, China in December 2019, SARS-CoV-2 spread rapidly and the World Health Organization (WHO) declared COVID-19 a pandemic on March 11, 2020 (1). This virus rapidly spread across the globe, infecting over 770 million people and causing more than 7 million deaths by the end of 2024 (2). The COVID-19 pandemic had a strong impact in the Andean Community, with an overall number of 13.212.404 cases and 422.144 deaths for Colombia, Ecuador, Peru, and Bolivia by the end of 2024 (Table 1). The WHO declared the end of the global health emergency of COVID-19 on May 5th 2023, after 3 years of profound disruption of the global economy and public health systems (3). Nevertheless, SARS-CoV-2 has not been eradicated and it is now another respiratory virus with seasonal outbreaks included in respiratory viruses’ surveillance programs alongside with influenza or respiratory syncytial virus (4).

**Table 1:** The burden of COVID-19 pandemic in Colombia, Ecuador, Peru, and Bolivia (The data presented was collected from the COVID-19 submission tracker of GISAID database for November 28th 2024).

Country	Number of COVID-19 cases	Number of COVID-19 deaths
Colombia	6,394,466	142,727
Peru	4,526,977	220,975
Bolivia	1,212,156	22,387
Ecuador	1,078,805	36,055
Total	13,212,404	422,144

SARS-CoV-2 has a large single-stranded RNA genome of approximately 30,000 nucleotides (5). As an RNA virus like influenza, SARS-CoV-2 has a high frequency of mutation that caused a rapid evolution and emergence of multiple lineages along COVID-19 pandemic. Emerging lineages have an improved transmissibility and also affect vaccines effectiveness or diagnostic tools accuracy (6). SARS-CoV-2 genomic surveillance allowed real time tracking of the viral evolution during COVID-19 pandemic, mapping the geographic spread of new variants of concern and identifying mutations that may increase transmissibility or confer immune evasion capabilities (5, 6). Since the beginning of the COVID-19 pandemic, numerous SARS-CoV-2 variants have emerged globally, characterized by distinct mutations profiles, and have been eventually replaced by new variants (6). The most important variants that rose concern during COVID-19 pandemic were B.1.1.7/20I/Alpha, B.1.351/20H/Beta, P.1/20 J/Gamma, B.1.617.2/21A/Delta and B.1.1.529/21 K;21 L/Omicron (the three nomenclatures used for each variant correspond to Pango/Nextclade/WHO classifications), either because an improved transmissibility, immune evasion, or disease severity (5–12). Since its emergence in late 2021, Omicron variant has been permanently evolving into multiple lineages that are still dominant worldwide (5, 6, 12). So far, SARS-CoV-2 genomic surveillance has been a fundamental tool for COVID-19 surveillance and control programs worldwide. In this sense, SARS-CoV-2 genomic surveillance represented the larger global effort for genomic sequencing of a pathogen ever done; for instance, during the first 2 years of COVID-19 pandemic, more than 10 million sequences were available via the EpiCoV database hosted by the Global Initiative on Sharing All Influenza Data (GISAID) initiative (13). By way of comparison, less than 2 million of influenza virus sequences were available for the period of 2008 till the second year of COVID-19 pandemic (13). Despites regional disparities depending on countries’ income, SARS-CoV-2 surveillance efforts have been made even at low and middle income countries (LMICs) as never seen before (13).

The Latin American and Caribbean Region has not been an exception in the global efforts to improve genomic surveillance of SARS-CoV-2. For instance, the Pan American Health Organization (PAHO) created the COVID-19 Genomic Surveillance Regional Network in 2020 to strengthen the sequencing capacity in national reference laboratories across the region (14). The establishment of this network has strengthened laboratory response capacity at the country level, as well as facilitated timely release of SARS-CoV-2 genomic information to be used to complement the multiple response strategies for COVID-19 pandemic mitigation; for instance, timely generation of SARS-CoV-2 genomic data is critical to support the development of diagnostic protocols and the information for vaccine development (14). The structure of this network includes a combination of in-country sequencing countries and those sending out for external sequencing to reference regional laboratories. Also, regional and country-level trainings were provided to generate timely information of SARS-CoV-2 genomic sequencing data shared through GISAID platform. This network included 8 regional reference sequencing laboratories located in Brazil, Chile, Colombia, Costa Rica, Mexico, Panama, Trinidad and Tobago, and the Centers for Disease Control and Prevention of USA to provide support for SARS-CoV-2 genome sequencing to all the countries members of PAHO (14). According to COVID-19 Genomic Surveillance Regional Network website, 591,977 virus sequences from Latin America and the Caribbean Member States of the PAHO have been generated by November 2023.^1^

Nevertheless, the latest one has not been the only regional initiative to support SARS-CoV-2 genomic surveillance in Latin America. The Andean Community currently includes the countries of Colombia, Ecuador, Peru y Bolivia. This community have developed several institutions to promote integration and collaboration at multiple levels between the country members, from economical to educational programs. In this context, to promote the integration of public health policies within the Andean Region, the “Convenio Hipólito Unanue” was created in the 70s and since them have evolved into the current “Organismo Andino de Salud-Convenio Hipólito Unanue” (ORAS-CONHU). The aim of ORAS-CONHU is to coordinate and support regional efforts within the Andean countries to improve people’s health. For instance, ORAS-CONHU coordinates meetings of the public health authorities of every country member to discuss regional plans for public health policies; it also promotes a coordinated “Andean response” to future health crisis through a network of regional institutions including the national health institutes of every country member. In this sense, and within the context of COVID-19 pandemic, ORAS-CONHU has led a regional program to promote SARS-CoV-2 genomic surveillance within the Andean Region including the national institutes of health in Colombia, Ecuador, Peru, and Bolivia that started on March 2022. The aim of this program has been to strengthen SARS-CoV-2 genomic surveillance’s regional capacity to support public health policies related to COVID-19 surveillance and control in the Andean Region. Another key objective was the creation of the Regional Observatory of Genomic Surveillance that, starting from SARS-CoV-2, could be eventually extended to other pathogens like dengue virus. This institution has been promoting an integrative SARS-CoV-2 genomic surveillance in the Andean Region with actions like laboratory and bioinformatics trainings, and regular meetings to share expertise and data between the national institutes of health of the four countries included in this program. Moreover, it has also supported the scientific knowledge by allocating funds to research projects and publications related to SARS-CoV-2 genomic surveillance in the Andean Region.

There are several studies that have provided a critical understanding into the genomic evolution and epidemiology of SARS-CoV-2 in Latin American Region, highlighting the co-circulation of multiple SARS-CoV-2 lineages and the emergence of variants of concern during COVID-19 pandemic (5, 10, 15–23). Moreover, some of those studies have been done in countries of the Andean Community like Colombia (18), Ecuador (19), and Peru (20, 21). Continuing those pioneer studies, this current study integrates genomic information for Colombia, Ecuador, Peru, and Bolivia for the period 2020–2024, that means during COVID-19 pandemic and post pandemic phase, to study the evolution of SARS-CoV-2 in the Andean Community. This study was carried out as a collaborative regional scientific project within the “ORAS-CONHU” regional program to promote SARS-CoV-2 genomic surveillance in the Andean Community.

This study aims to characterize the Andean Community efforts in terms of SARS-CoV-2 genomic surveillance and to address the evolution and variants diversity of SARS-CoV-2 in those countries since the initial outbreak of COVID-19 till the end of 2024.

## Materials and methods

### Data retrieval

Complete SARS-CoV-2 whole-genome sequences from COVID-19 cases in Bolivia, Colombia, Ecuador, and Peru were obtained from the GISAID platform (accessed on 1 February 2025).^2^

To evaluate the sequencing efforts across Colombia, Peru, Ecuador and Bolivia, data on the total number of confirmed cases and SARS-CoV-2 genomes sequenced (101,740) were collected for each country for February 1st 2020 to December 31st 2024.

### SARS-CoV-2 emerging lineages distribution, clade determination, and phylogenetic analysis

**Figure 1:**
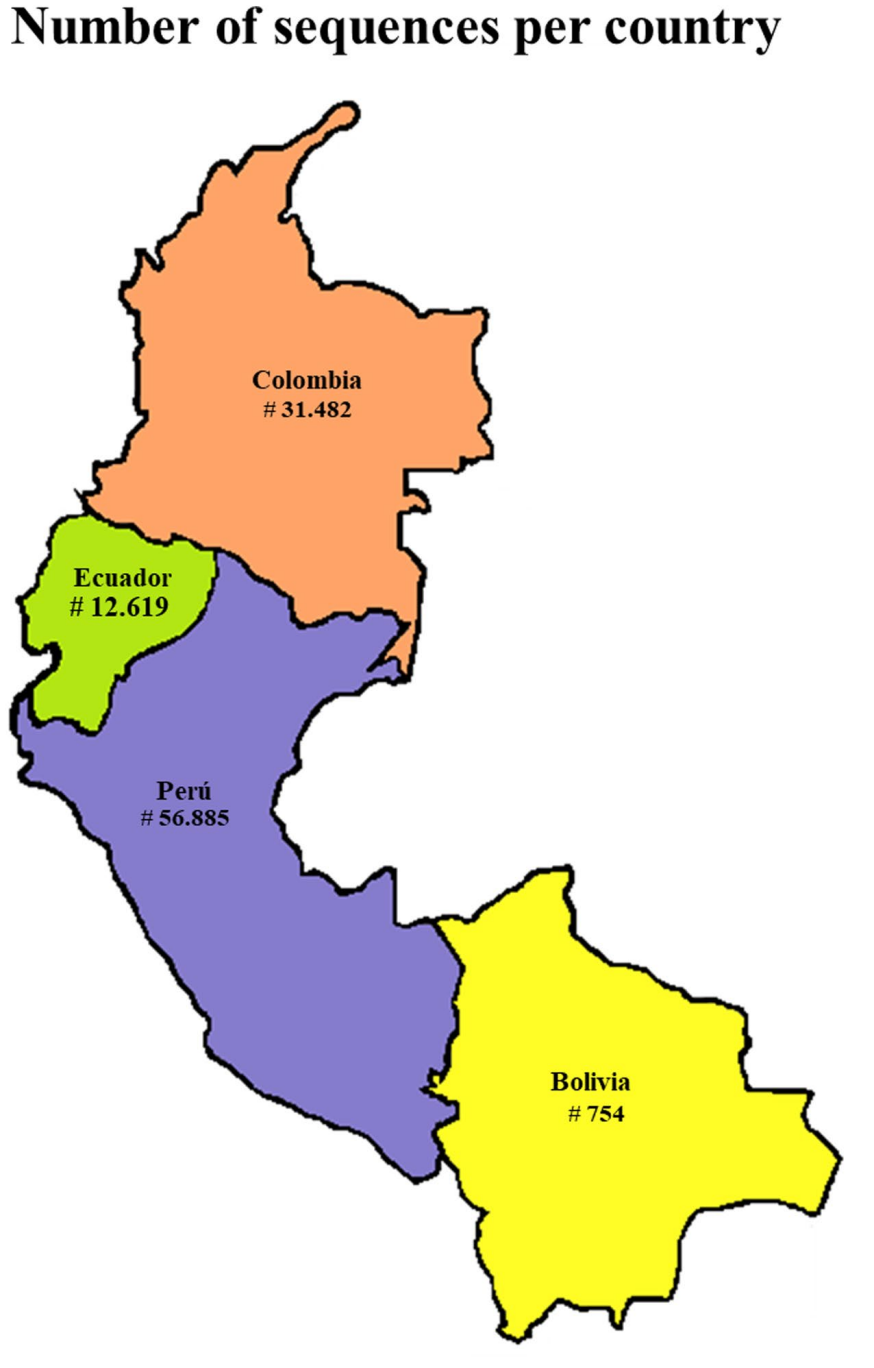
Map illustrating the Andean countries of Colombia, Ecuador, Peru, and Bolivia included in this study and the total number of SARS-CoV-2 genomes generated for each country.

To be included in these analysis, genomes were required to exceed 29,000 nucleotides in length and had to be submitted between 1 January 2020 and 31 December 2024. The SARS-CoV-2 reference genome, Wuhan-Hu-1, was also retrieved from the NCBI database (Accession Number: NC_045512.2). Due to the large volume of sequenced genomes available in GISAID, a representative subset of 16,867 genomes from Andean countries (Bolivia, Colombia, Ecuador, and Peru) was selected using the “subsample-max-sequence” function in the Nextstrain workflow, prioritizing genomes with higher genetic divergence. Sampling criteria included metadata filters for country of origin, collection date, and minimum sequence length, ensuring temporal and geographic representativeness. The subsampling strategy selected sequences that maximize genetic diversity by iteratively choosing those most divergent from previously selected ones, based on pairwise comparisons. To ensure reproducibility, the workflow was executed using the augur filter tool with a fixed random seed and consistent filtering parameters. This guarantees that the same input dataset and configuration will yield identical outputs. While this approach enhances phylogenetic resolution, it may underrepresent recent transmission clusters or epidemiologically linked cases, introducing potential bias in lineage frequency and geographic spread analyses (Figure 1).

**Figure 2:**
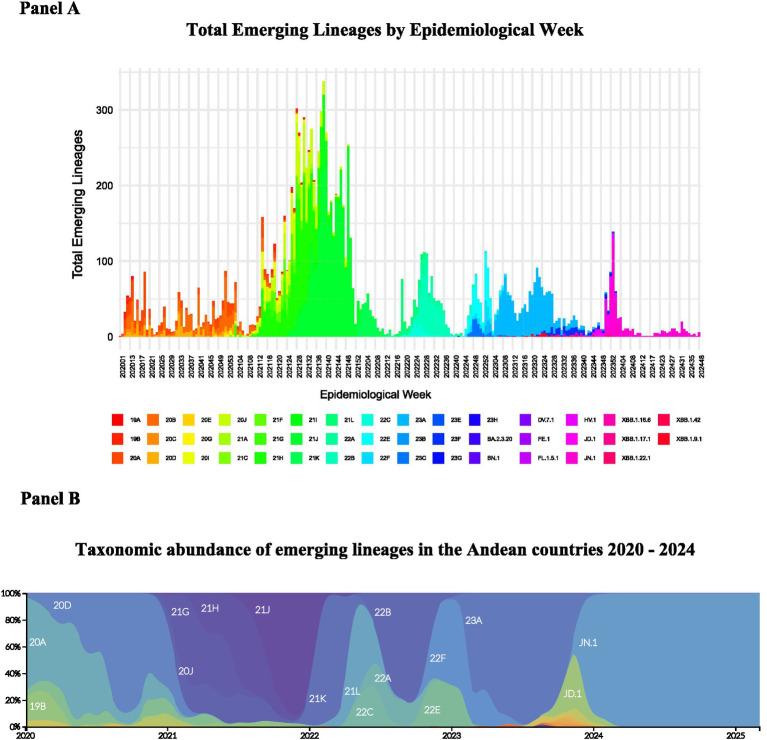
SARS-CoV-2 emerging lineages and clades distribution in Colombia, Ecuador, Peru, and Bolivia during 2020–2024. **(A)** Total emerging lineages by epidemiological week. **(B)** Taxonomic abundance of SARS-CoV-2 clades.

Emerging lineages distribution by epidemiological week (Figure 2A) were analyzed using RStudio 2023.06.1 + 524 with R 4.3.1 and represented using Nextclade nomenclature. The dataset was processed with the ggplot2 and dplyr libraries. Data were grouped by epidemiological week (epiweek) and emerging lineage (emerging_lineage), and the total sequences count was calculated. A stacked bar chart was created to visualize the distribution of lineages across weeks, with custom colors for each lineage. The x-axis labels were rotated for readability, and only selected weeks were shown.

**Figure 3:**
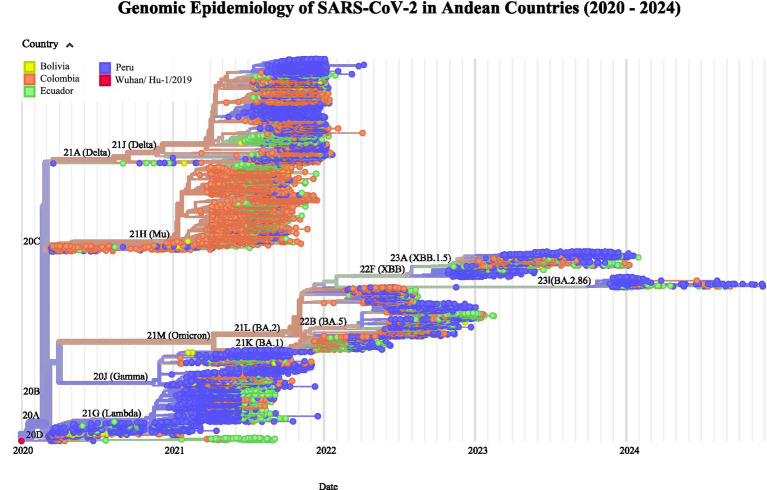
Maximum likelihood phylodynamic analysis of SARS-CoV-2 in Colombia, Ecuador, Peru, and Bolivia during 2020–2024 (The root of the phylogenetic tree is represented by the Wuhan/Hu-1/2019 sequence).

Clade determination and phylogenetic analysis were performed using the Nextstrain platform (accessed February 18, 2025)^3^ (24). The Nextstrain workflow was executed using the augur pipeline, which included sequence alignment, maximum likelihood phylodynamic analysis to estimate temporal evolution and pathogen population dynamics, and temporal dating of ancestral nodes. A discrete trait geographic reconstruction was also performed to map the genomes according to their geographical distribution across the study countries, enabling the identification of patterns in pathogen spread. Finally, phylogenetic results were visualized using Nextstrain’s graphical tools, facilitating the interpretation of evolutionary relationships and geographic propagation of the virus over time (Figures 2B, 3).

## Results

### SARS-CoV-2 sequencing efforts in Colombia, Ecuador, Peru, and Bolivia

The number of SARS-CoV-2 sequences for each country is detailed in Table 2. Overall, 101,740 SARS-CoV-2 sequences were available for the period 2020–2024. For Colombia, a total number of 31,482 was obtained for the period of January 1st 2020 to December 31st 2024, distributed as 773 for 2020, 14,626 for 2021, 12,427 for 2022, 3,174 for 2023 and 426 for 2024. For Ecuador, a total number of 12,619 was obtained for the period of January 1st 2020 to December 31st 2024, distributed as 814 for 2020, 4,018 for 2021, 5,102 for 2022, 1,796 for 2023, and 744 for 2024. For Peru, a total number of 56,855 was obtained for the period of January 1st 2020 to December 31st 2024, distributed as 1,382 for 2020, 15,228 for 2021, 32,250 for 2022, 6,285 for 2023, and 1,914 for 2024. For Bolivia, a total number of 754 was obtained for the period of January 1st 2020 to December 31st 2024, distributed as 93 for 2020, 306 for 2021, 219 for 2022, 100 for 2023, and 34 for 2024.

**Table 2:** SARS-CoV-2 Genomic surveillance in Colombia, Ecuador, Peru, and Bolivia.

Country	2020	2021	2022	2023	2024	total	% sequenced
Peru	1,382	15,228	32,250	6,275	1,914	56,885	1.25
Colombia	773	14,626	12,427	3,174	426	31,482	0.49
Ecuador	814	4,018	5,102	1,796	744	12,619	1.15
Bolivia	93	306	219	100	34	754	0.06
Total	3,062	34,178	49,998	11,345	3,118	101,740	0.77

Number of sequences uploaded to GISAID database for every country and year is shown (% sequenced: percentage of COVID-19 cases sequenced for every country).

As it is detailed in Table 2, the percentage of SARS-CoV-2 cases that was sequenced in every country was 0.49% for Colombia, 1.15% for Ecuador, 1.25% for Peru and 0.06% for Bolivia.

### SARS-CoV-2 emerging lineages distribution, clade determination and phylogenetic analysis in Colombia, Ecuador, Peru, and Bolivia during 2020–2024

For the SARS-CoV-2 emerging lineages distribution, clade determination and phylogenetic analysis, 16,867 genomes of SARS-CoV-2 for the period 2020–2024 were selected as detailed in the methods. The SARS-CoV-2 emerging lineages distribution by epidemiological week is detailed in Figure 2A while the SARS-CoV-2 clade distribution is illustrated in Figure 2B. In Figure 3, the phylogenetic analysis of SARS-CoV-2 sequences displaying different colors for Colombia, Ecuador, Peru, and Bolivia is shown. The root of the phylogenetic tree is represented by the Wuhan/Hu-1/2019 sequence.

From the initial lineages 19A and 19B, the region experienced a rapid diversification of lineages like elsewhere, as demonstrated by the phylogenetic analysis conducted using the Nextstrain tool. In 2020, lineages 20A, 20B, and 20C emerged, characterized by key mutations in the spike protein which enhanced viral transmissibility. In 2021, several variants such as 20 J (Gamma), 21A (Delta), 21G (Lambda), and 21H (Mu) emerged, each with distinct transmissibility and public health impact. This was followed by the emergence of the highly transmissible Omicron variants (21 K, 21 L) at the end of 2022 that has been the dominant variant since then but evolving into multiple sub variants lineages like 22F, 23A, and 23I. This last lineage 23I (JN.1) has been dominant along 2024.

So far, the relative taxonomic abundance of various emerging SARS-CoV-2 lineages over time is displayed in Figure 2B, where each color in the graph corresponds to a specific viral lineage, and the shaded areas show how the prevalence of these lineages has shifted over the study period. This figure enables visualization of viral evolutionary dynamics, highlighting the emergence, increased frequency, or extinction of certain lineages in response to selective pressure, public health measures, and population immunity. Figure 2B shows the diversity of circulating lineages and underscores the current dominance of the 23I (JN.1) lineage, which has exhibited greater abundance compared to other emerging lineages, showcasing its evolutionary success.

The phylogenetic analysis in Figure 3 shows the SARS-CoV-2 evolutionary trend for Colombia, Ecuador, Peru, and Bolivia. While each country exhibits some specific characteristics, the phylogenetic tree reflects a common pattern in the variability and evolution of SARS-CoV-2 for those four countries underscoring the rapid transnational transmission of the virus.

## Discussion

Our study shows an uneven distribution of genomic surveillance efforts across the Andean countries of Colombia, Ecuador, Peru, and Bolivia. The larger number of sequences came from Peru, followed by Colombia, Ecuador, and Bolivia. However, when this data was normalized with the burden of COVID-19 cases in every country, we obtained that 1, 25, 1.15, 0.49, and 0.06% of cases were sequenced in Peru, Ecuador, Colombia, and Bolivia. Those results show that SARS-CoV-2 sequencing capacities in Bolivia were clearly below its neighbor countries. Notably, both Ecuador and Peru achieved the 1% threshold of total COVID-19 cases sequenced, an important milestone for LMICs (13). This regional imbalance in SARS-CoV-2 genomic surveillance has also been observed globally, driven by socioeconomic factors and pre-pandemic laboratory and surveillance capacities (13). In fact, Bolivia is the lowest income country from the Andean Community, and that would explain its lower sequencing capacity compared to Ecuador, Peru, and Colombia. Similar findings have been reported in a recent study from Brazil, where strong differences in resources allocated to sequencing efforts were found between different states regardless of their COVID-19 pandemic burden (5). So far, there are strong disparities in SARS-CoV-2 sequencing capacities in the countries of the Andean Community as it has already been described for Latin American countries (14, 15) and worldwide (13). We emphasize the essential role of collaborative networks and local, regional and international funding in bolstering more equity in genomic surveillance capacities across the countries in the Andean Community by specifically promoting those countries with lower sequencing capacities like Bolivia. Key institutions for each country like the national institutes of health or equivalent, in partnership with PAHO or ORAS-CONHU could play a pivotal role in developing an integrative regional sequencing strategy for the Andean countries. In fact, as it has been described in this study, these collaborative efforts have already been fundamental in strengthening technical capacities, facilitating data sharing, and promoting genomic sequencing in LMICs of the Americas as previously reported by PAHO and now for us. In this sense, our colleagues from Bolivia could learn from the successful experiences from their neighbor countries to improve its genomic surveillance capacities beyond SARS-CoV-2. So far, an uneven sequencing capacity could potentially impact the success of integrated regional or global SARS-CoV-2 surveillance strategies, and new variants could appear anywhere. Timely detection of SARS-CoV-2 variants worldwide is crucial for an effective global surveillance and control strategy. As seen with unequal distribution of SARS-CoV-2 vaccines, unequal genomic surveillance in LMICs could jeopardize genomic sequencing efforts in developed countries.

Regarding the SARS-CoV-2 variants distribution and phylogeny in 2020–2024, our results revealed an expected notable diversity of viral lineages since the beginning of COVID-19 pandemic, with a sequential emergence of new variants, including some variants of regional interest like 21G (lambda, or so-called “Andean variant”), as it has already been reported for Latin American countries (5, 10, 15–17, 22) and also for countries included in our study like Ecuador (18), Colombia (19) and Peru (20, 21). So far, our study not only endorses these previous reports and shares the collaborative experience of the ORAS-CONHU program; it also provides an updated perspective up SARS-CoV-2 evolution till the end of 2024, showing the current dominance of omicron derived lineages like the 23I (JN.1) lineage (25). Moreover, our phylogenetic analysis also shows a regional trend of SARS-CoV-2 evolution for the Andean Region supporting the idea of a Regional Observatory for Genomic Surveillance of SARS-CoV-2 promoted by ORAS-CONHU. Future steps for this sustainable and integrated surveillance network in the Andean Community could include a common budget to guarantee an equal distribution of sequencing across countries, to support laboratory and bioinformatics trainings for network members and promote regular meetings to share expertise and data between the stakeholders of the four countries included in this current ORAS-CONHU program. Moreover, the experience gained with SARS-CoV-2 genomic surveillance could set the grounds for a future Andean genomic surveillance program of infectious diseases including other pathogens like tuberculosis, respiratory viruses like respiratory syncytial virus or influenza virus (with special attention to H5N1 or other avian flu viruses), arboviruses including dengue but other emerging ones like yellow fever or chikungunya viruses, or other neglected and endemic diseases to the region like leptospirosis or trypanosomiasis.

Our study has some limitations that we would like to acknowledge. First, only the four countries within the Andean Community were included in the study, although the Andean Region includes also Chile and Venezuela; in this sense, future regional surveillance programs should encourage also other neighbor countries in the Andean Region like Chile and Venezuela, or even across Latin America, to join. Second, some methodological bias should be considered. In particular, potential confounders such as testing rates, sequencing technologies, and metadata quality were not thoroughly addressed. Variability in testing coverage may affect the representativeness of sequenced samples, while differences in sequencing platforms and protocols can influence mutation detection and lineage assignment. Moreover, incomplete or inconsistent metadata—especially regarding collection dates and geographic origin—may introduce uncertainty in phylogenetic and temporal analyses.

In conclusion, this study provides a detailed overview of the molecular evolution of SARS-CoV-2 in the Andean countries of Colombia, Ecuador, Peru, and Bolivia from COVID-19 outbreak in 2020 to the post pandemic phase in 2024, emphasizing the importance of monitoring the genetic diversity of the virus to support effective control and prevention strategies. The analysis of GISAID database revealed significant regional disparities in SARS-CoV-2 sequencing efforts, highlighting the need to specifically improve genomic surveillance in Bolivia to keep up an effective genomic tracking of new variants in the Andean Region. The lessons learned from this study will help ORAS-CONHU to lead future efforts to keep improving a regional network for genomic surveillance of emergent and neglected pathogens, or future pandemics.

## Data Availability

The original contributions presented in the study are included in the article/supplementary material, further inquiries can be directed to the corresponding author.

## Footnotes

^1^ https://www.paho.org/en/topics/influenza-sars-cov-2-rsv-and-other-respiratory-viruses/resvigen-respiratory-virus-genomic

^2^ https://www.gisaid.org/

^3^ https://nextstrain.org/ncov

## Grupo Andino de Vigilancia Genómica de SARS-CoV-2

María del Carmen Calle Dávila, Marisela Mallqui Osorio, Luis Beingolea More, Walter Vigo Valdez and Alondra Tribeños Palomino from Organismo Andino de Salud–Convenio Hipólito Unanue (ORAS-CONHU), Lima, Perú; Dioselina Peláez-Carvajal, Martínez Vargas Daniel, Alicia Rosales, Beatriz Elena De arco-Rodriguez, Jhindy Tatiana Perez-Lozada, Jaime Alexander Chivatá-Ávila, Natalya Barandica, Tatiana Cobos, Katherine Laiton-Donato, Diego A. Álvarez-Díaz from Grupo de Genómica de Microorganismos Emergentes, Dirección de Investigación en Salud Pública, Instituto Nacional de Salud, Bogotá, Colombia; Denisses Portugal and Lourdes Pardo from Insituto Nacional de Salud Pública e Investigación, Ecuador.

## References

[1] Wang C Horby PW Hayden FG Gao GF. A novel coronavirus outbreak of global health concern. Lancet. (2020) 395:470–3. doi: 10.1016/S0140-6736(20)30185-9, PMID: 31986257 PMC7135038

[2] Peacock TP Penrice-Randal R Hiscox JA Barclay WS. SARS-CoV-2 one year on: evidence for ongoing viral adaptation. J Gen Virol. (2021) 102:001584. doi: 10.1099/jgv.0.001584, PMID: 33855951 PMC8290271

[3] World Health Organization. Statement on the fifteenth meeting of the IHR (2005) emergency committee on the COVID-19 pandemic WHO director general’s speeches. Geneva: World Health Organization (2023).

[4] World Health Organization. End-to-end integration of SARS-CoV-2 and influenza sentinel surveillance: compendium of country approaches. Geneva: World Health Organization (2023).

[5] Souza UJBd Spilki FR Tanuri A Roehe PM Campos FS. Two years of SARS-CoV-2 omicron genomic evolution in Brazil (2022–2024): subvariant tracking and assessment of regional sequencing efforts. Viruses. (2025) 17:64. doi: 10.3390/v17010064, PMID: 39861853 PMC11768930

[6] Angius F Puxeddu S Zaimi S Canton S Nematollahzadeh S Pibiri A. SARS-CoV-2 evolution: implications for diagnosis, treatment, vaccine effectiveness and development. Vaccine. (2025) 13:17. doi: 10.3390/vaccines13010017 PMC11769326 39852796

[7] Rambaut A Holmes EC O’Toole Á Hill V McCrone JT Ruis C. A dynamic nomenclature proposal for SARS-CoV-2 lineages to assist genomic epidemiology. Nat Microbiol. (2020) 5:1403–7. doi: 10.1038/s41564-020-0770-5, PMID: 32669681 PMC7610519

[8] Li J Lai S Gao GF Shi W. The emergence, genomic diversity and global spread of SARS-CoV-2. Nature. (2021) 600:408–18. doi: 10.1038/s41586-021-04188-6, PMID: 34880490

[9] Araf Y Akter F Tang Y Fatemi R Parvez MSA Zheng C. Omicron variant of SARS-CoV-2: genomics, transmissibility, and responses to current COVID-19 vaccines. J Med Virol. (2022) 94:1825–32. doi: 10.1002/jmv.27588, PMID: 35023191 PMC9015557

[10] Giovanetti M Slavov SN Fonseca V Wilkinson E Tegally H Patané JSL. Genomic epidemiology of the SARS-CoV-2 epidemic in Brazil. Nat Microbiol. (2022) 7:1490–500. doi: 10.1038/s41564-022-01191-z, PMID: 35982313 PMC9417986

[11] de Souza UJB dos Santos RN Campos FS Lourenço KL da Fonseca FG Spilki FR. High rate of mutational events in SARS-CoV-2 genomes across Brazilian geographical regions, February 2020 to June 2021. Viruses. (2021) 13:1806. doi: 10.3390/v13091806 34578387 PMC8473193

[12] Planas D Staropoli I Michel V Lemoine F Donati F Prot M. Distinct evolution of SARS-CoV-2 omicron XBB and BA.2.86/JN.1 lineages combining increased fitness and antibody evasion. Nat Commun. (2024) 15:2254. doi: 10.1038/s41467-024-46490-7, PMID: 38480689 PMC10938001

[13] Brito AF Semenova E Dudas G Hassler GW Kalinich CC Kraemer MUG. Global disparities in SARS-CoV-2 genomic surveillance. Nat Commun. (2022) 13:7003. doi: 10.1038/s41467-022-33713-y, PMID: 36385137 PMC9667854

[14] Leite JA Vicari A Perez E Siqueira M Resende P Motta FC. Implementation of a COVID-19 genomic surveillance regional network for Latin America and Caribbean region. PLoS One. (2022) 17:e0252526. doi: 10.1371/journal.pone.0252526, PMID: 35239677 PMC8893691

[15] Ribeiro Dias MF Andriolo BV Silvestre DH Cascabulho PL Leal da Silva M. Genomic surveillance and sequencing of SARS-CoV-2 across South America. Rev Panam Salud Publica. (2023) 47:1. doi: 10.26633/RPSP.2023.21, PMID: 36686893 PMC9847409

[16] Gräf T Martinez AA Bello G Dellicour S Lemey P Colizza V. Dispersion patterns of SARS-CoV-2 variants gamma, lambda and mu in Latin America and the Caribbean. Nat Commun. (2024) 15:1837. doi: 10.1038/s41467-024-46143-9, PMID: 38418815 PMC10902334

[17] Ortiz-Pineda PA Sierra-Torres CH. Evolutionary traits and genomic surveillance of SARS-CoV-2 in South America. Glob Health Epidemiol Genom. (2022) 2022:1–9. doi: 10.1155/2022/8551576, PMID: 35655960 PMC9132712

[18] Gutierrez B Márquez S Prado-Vivar B Becerra-Wong M Guadalupe JJ da Silva Candido D. Genomic epidemiology of SARS-CoV-2 transmission lineages in Ecuador. Virus Evol. (2021) 7:veab051. doi: 10.1093/ve/veab051 34527281 PMC8244811

[19] Jimenez-Silva C Rivero R Douglas J Bouckaert R Villabona-Arenas CJ Atkins KE. Genomic epidemiology of SARS-CoV-2 variants during the first two years of the pandemic in Colombia. Commun Med. (2023) 3:97. doi: 10.1038/s43856-023-00328-3, PMID: 37443390 PMC10344885

[20] Jimenez-Vasquez V Vargas-Herrera N Bárcena-Flores L Hurtado V Padilla-Rojas C Araujo-Castillo RV. Dispersion of SARS-CoV-2 lineage BA.5.1.25 and its descendants in Peru during two COVID-19 waves in 2022. Genom Inform. (2024) 22. doi: 10.1186/s44342-024-00006-3, PMID: 38907313 PMC11184951

[21] Vargas-Herrera N Araujo-Castillo RV Mestanza O Galarza M Rojas-Serrano N Solari-Zerpa L. SARS-CoV-2 lambda and gamma variants competition in Peru, a country with high seroprevalence. Lancet Reg Health. (2022) 6:100112. doi: 10.1016/j.lana.2021.100112, PMID: 34812432 PMC8600335

[22] Candido DS Claro IM de Jesus JG Souza WM Moreira FRR Dellicour S. Evolution and epidemic spread of SARS-CoV-2 in Brazil. Science. (2020) 369:1255–60. doi: 10.1126/science.abd2161, PMID: 32703910 PMC7402630

[23] Arantes I Gomes M Ito K Sarafim S Gräf T Miyajima F. Spatiotemporal dynamics and epidemiological impact of SARS-CoV-2 XBB lineage dissemination in Brazil in 2023. Microbiol Spectr. (2024) 12:e03831-23. doi: 10.1128/spectrum.03831-23, PMID: 38315011 PMC10913747

[24] Hadfield J Megill C Bell SM Huddleston J Potter B Callender C. Nextstrain: real-time tracking of pathogen evolution. Bioinformatics. (2018) 34:4121–3. doi: 10.1093/bioinformatics/bty407, PMID: 29790939 PMC6247931

[25] Looi M-K. Covid-19: WHO adds JN.1 as new variant of interest. BMJ. (2023) 383:p2975. doi: 10.1136/bmj.p2975, PMID: 38128957

